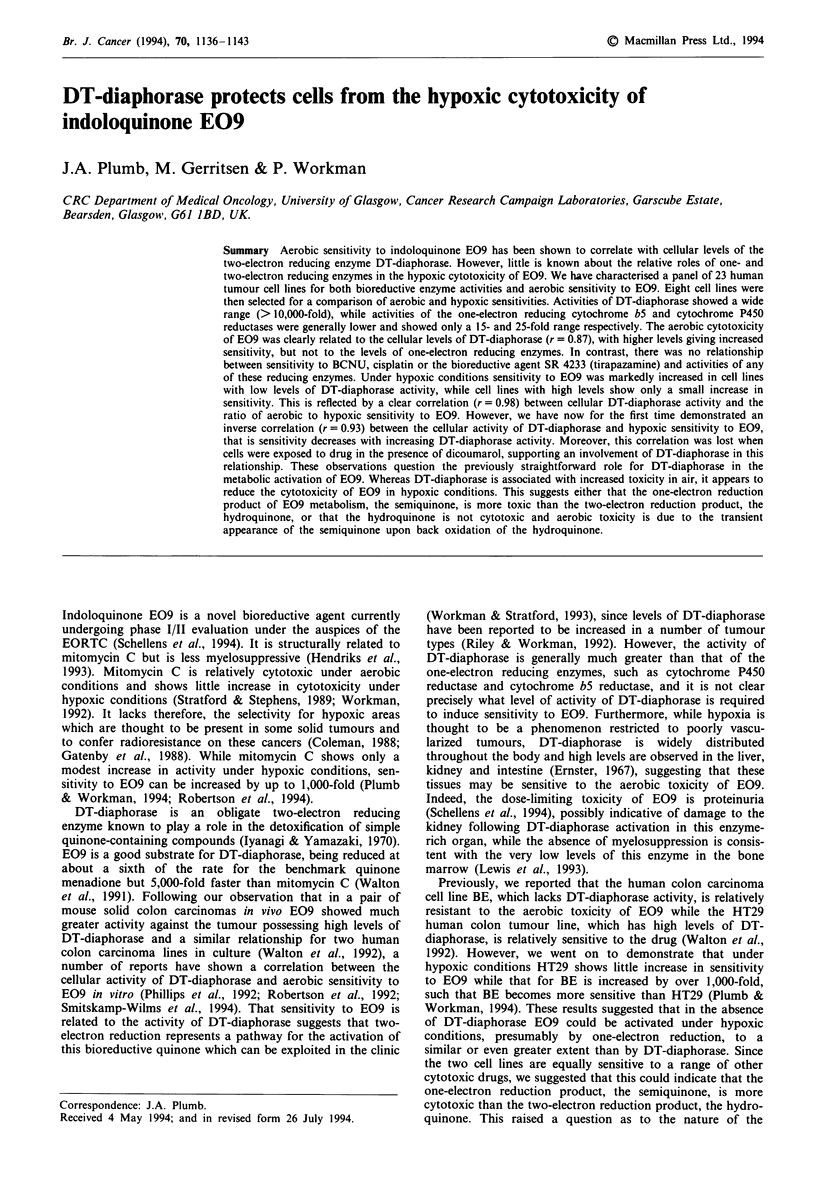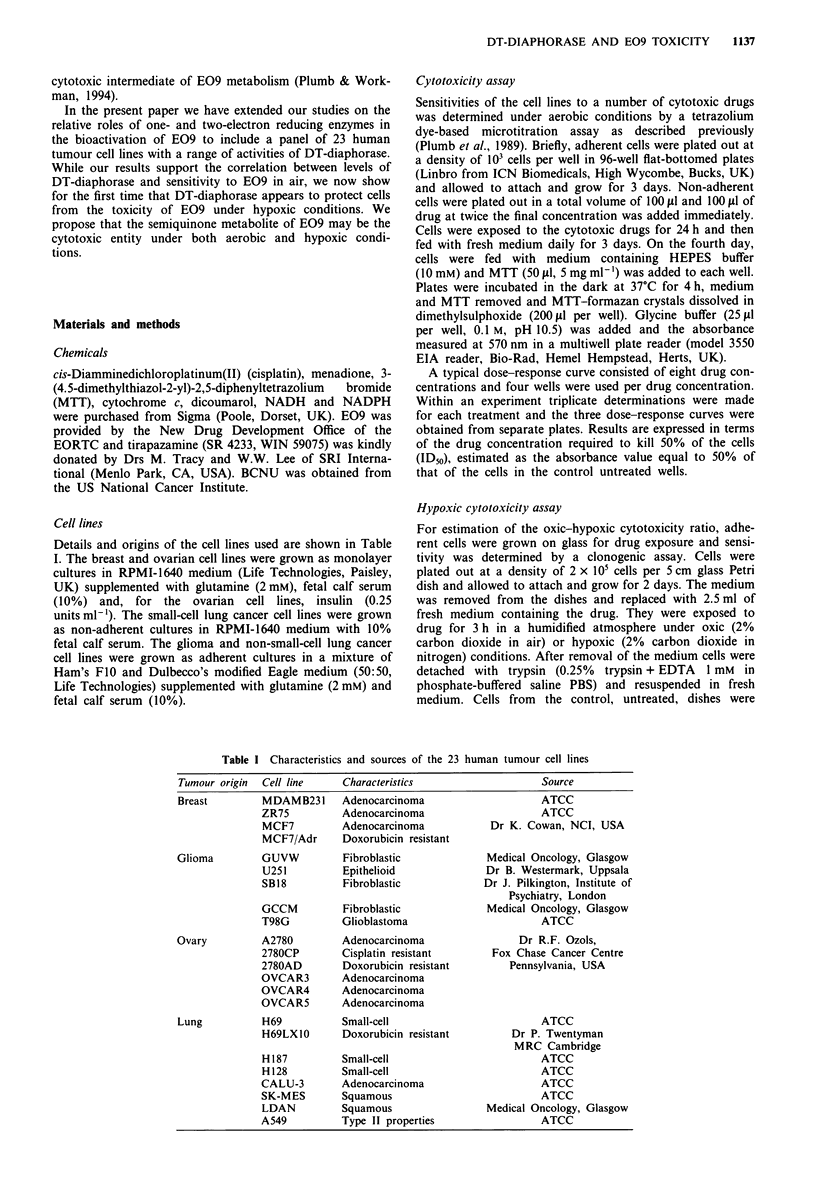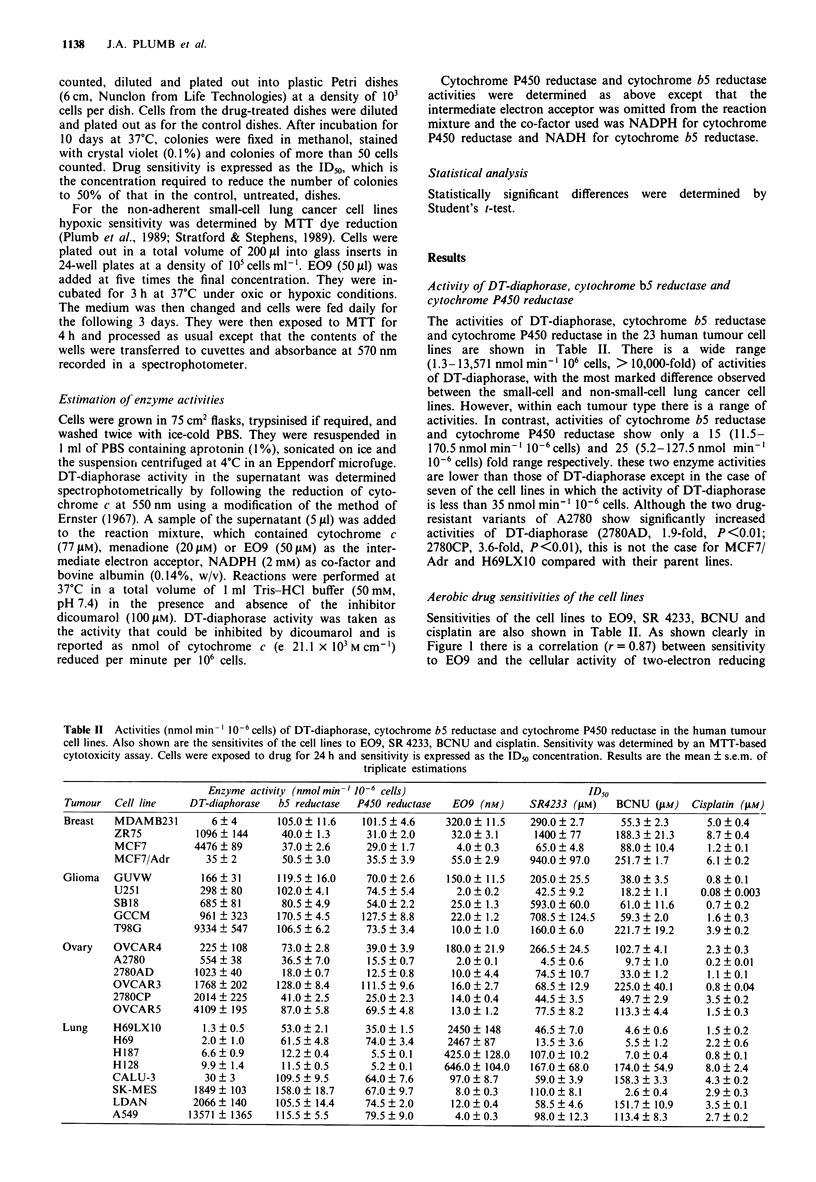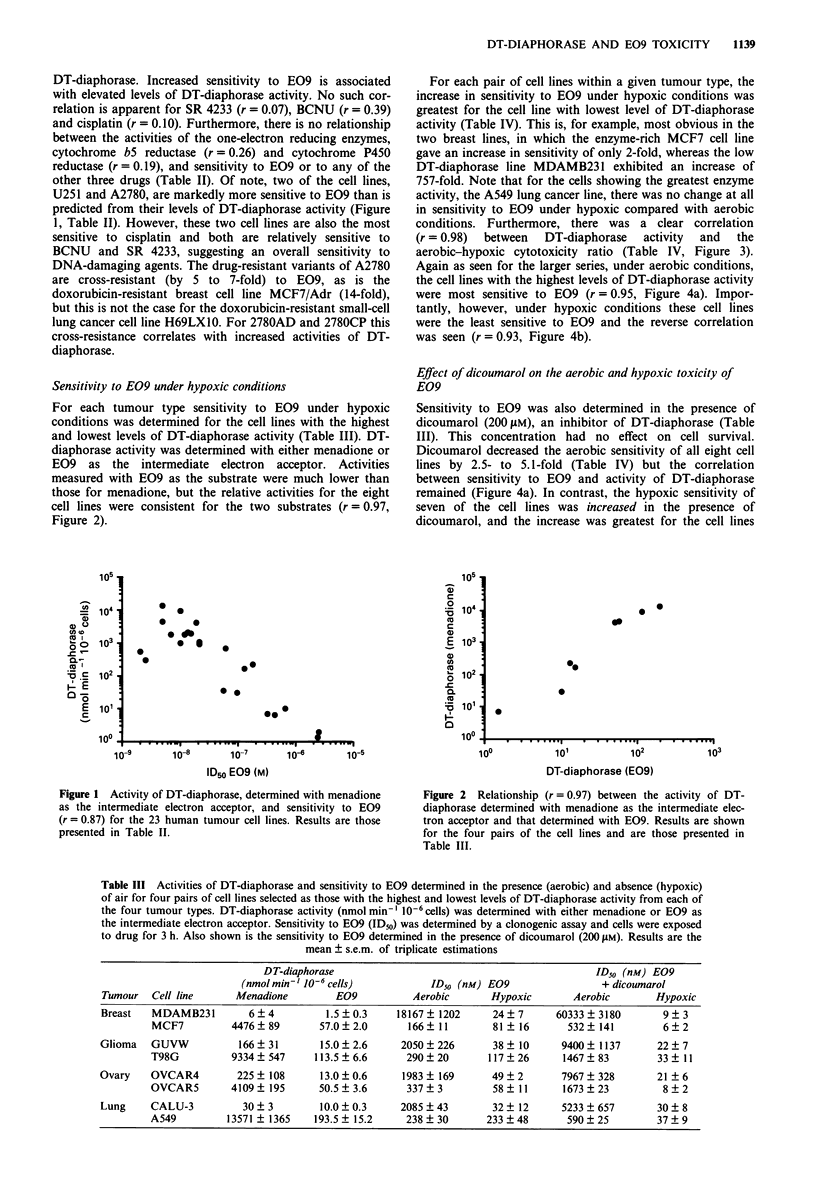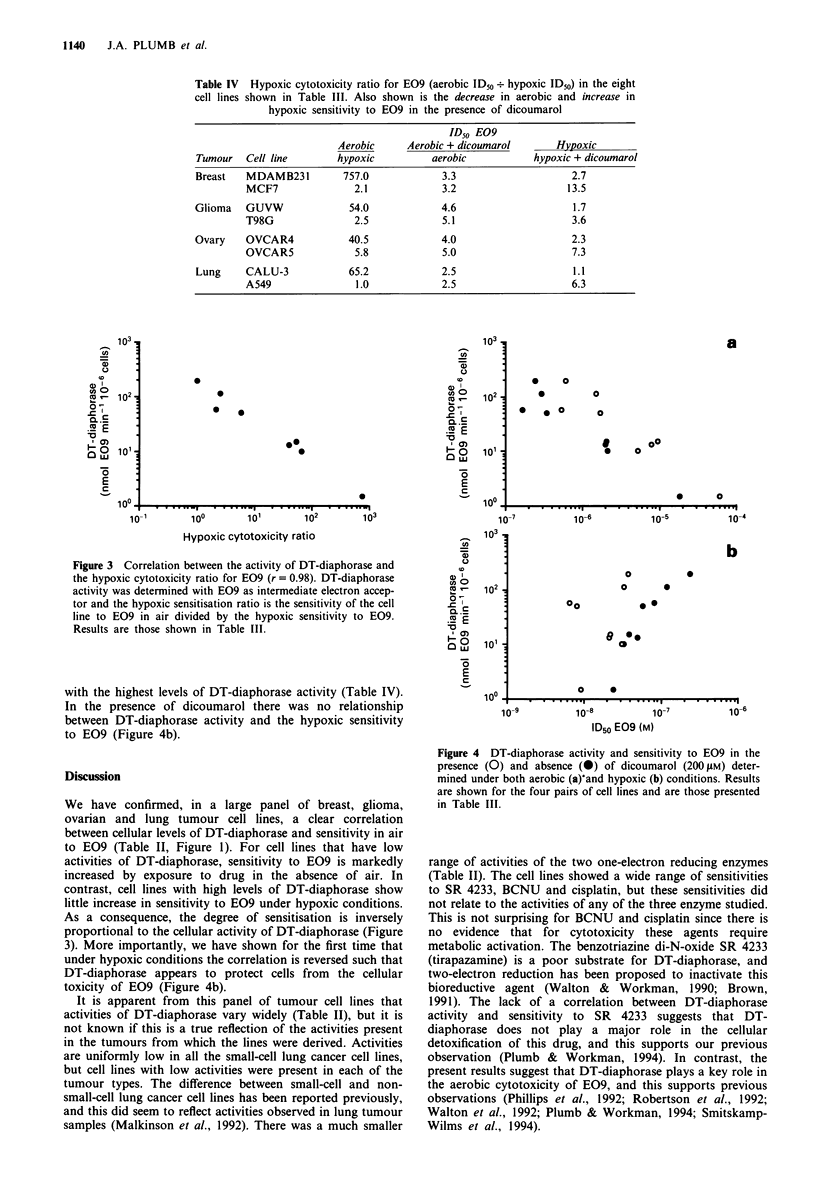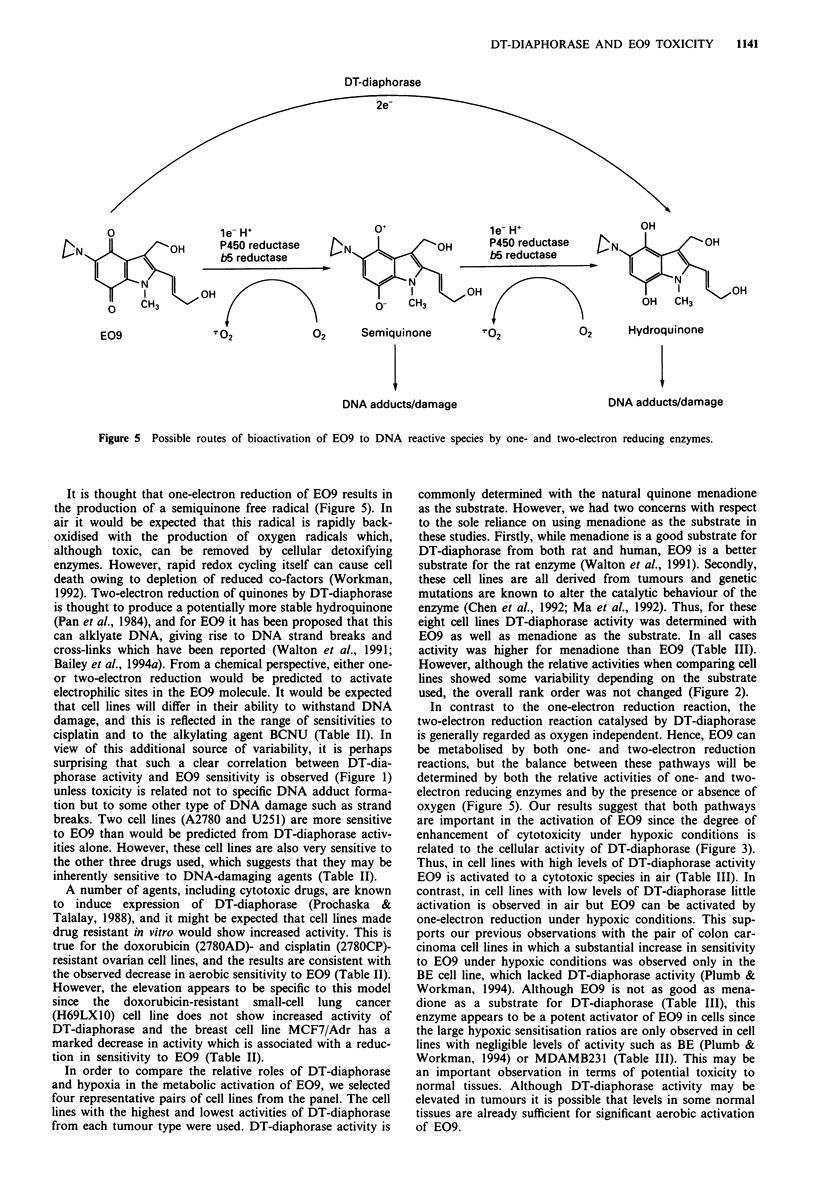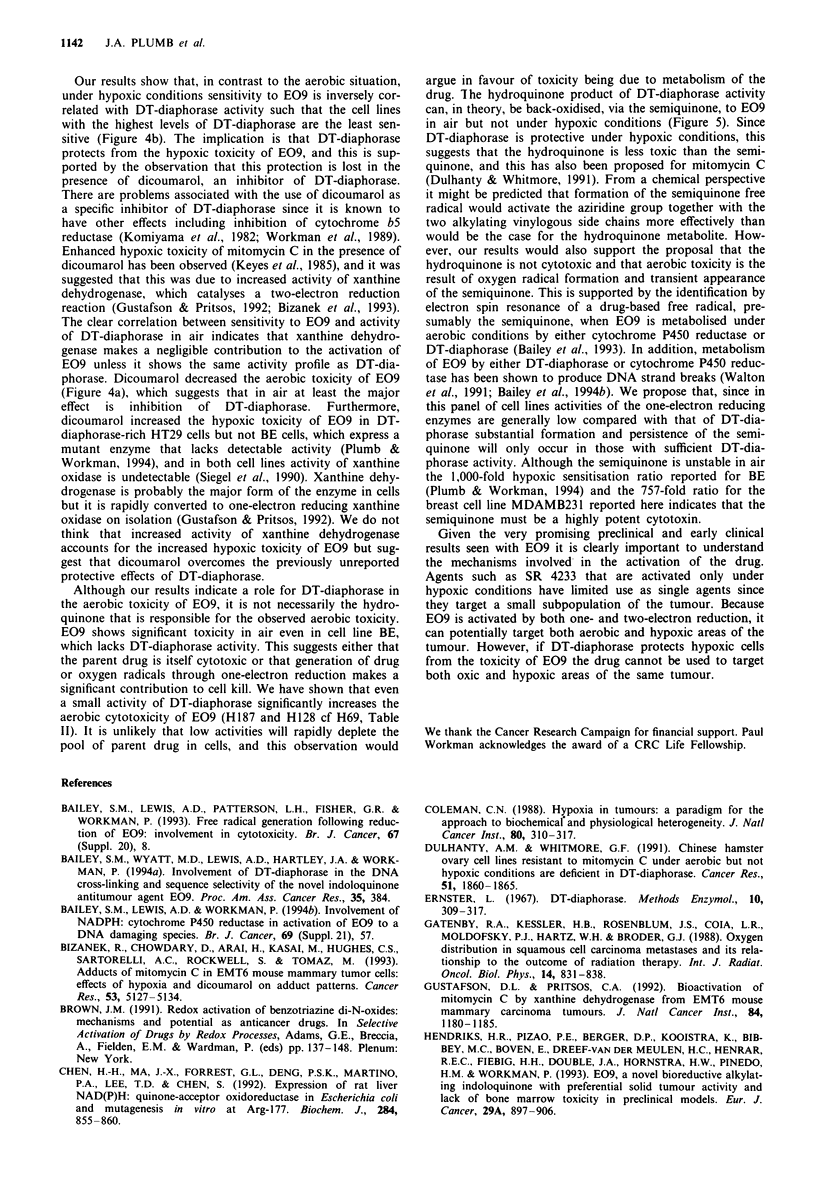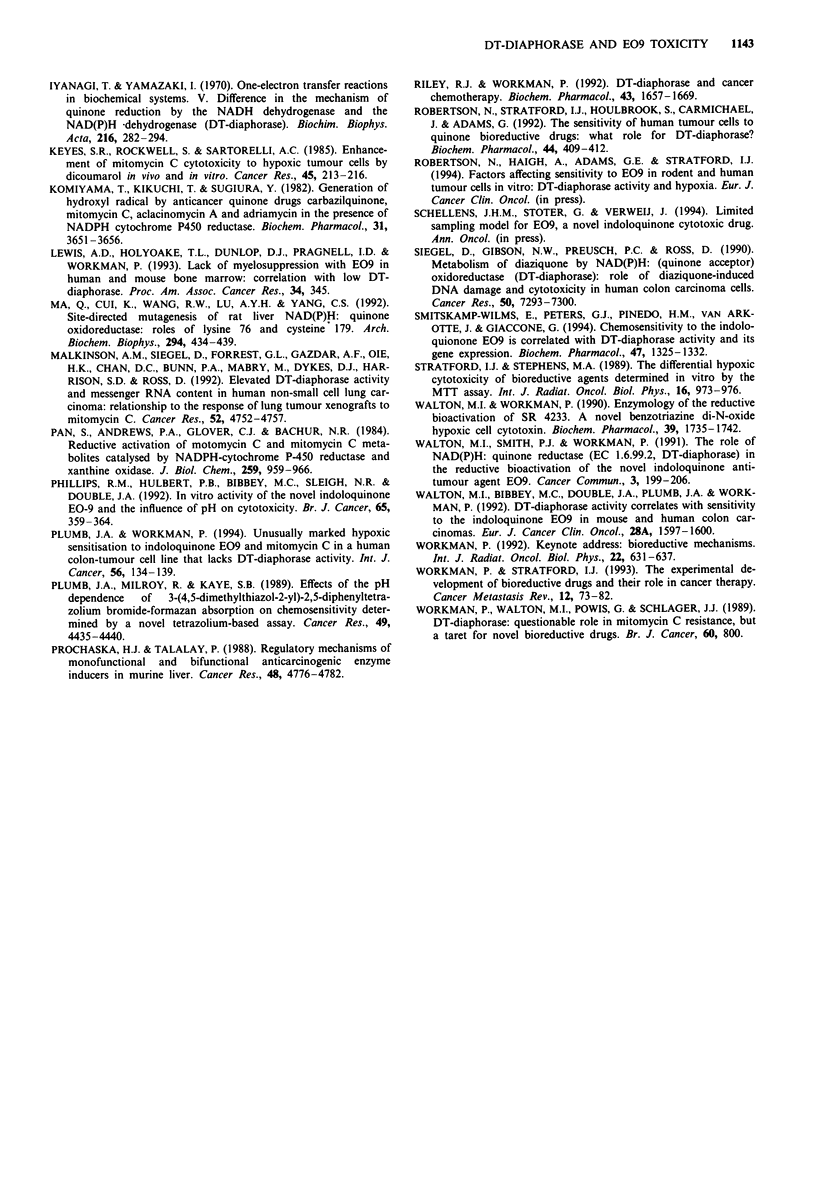# DT-diaphorase protects cells from the hypoxic cytotoxicity of indoloquinone EO9.

**DOI:** 10.1038/bjc.1994.461

**Published:** 1994-12

**Authors:** J. A. Plumb, M. Gerritsen, P. Workman

**Affiliations:** CRC Department of Medical Oncology, University of Glasgow, Bearsden, UK.

## Abstract

Aerobic sensitivity to indoloquinone EO9 has been shown to correlate with cellular levels of the two-electron reducing enzyme DT-diaphorase. However, little is known about the relative roles of one- and two-electron reducing enzymes in the hypoxic cytotoxicity of EO9. We have characterised a panel of 23 human tumour cell lines for both bioreductive enzyme activities and aerobic sensitivity to EO9. Eight cell lines were then selected for a comparison of aerobic and hypoxic sensitivities. Activities of DT-diaphorase showed a wide range (> 10,000-fold), while activities of the one-electron reducing cytochrome b5 and cytochrome P450 reductases were generally lower and showed only a 15- and 25-fold range respectively. The aerobic cytotoxicity of EO9 was clearly related to the cellular levels of DT-diaphorase (r = 0.87), with higher levels giving increased sensitivity, but not to the levels of one-electron reducing enzymes. In contrast, there was no relationship between sensitivity to BCNU, cisplatin or the bioreductive agent SR 4233 (tirapazamine) and activities of any of these reducing enzymes. Under hypoxic conditions sensitivity to EO9 was markedly increased in cell lines with low levels of DT-diaphorase activity, while cell lines with high levels show only a small increase in sensitivity. This is reflected by a clear correlation (r = 0.98) between cellular DT-diaphorase activity and the ratio of aerobic to hypoxic sensitivity to EO9. However, we have now for the first time demonstrated an inverse correlation (r = 0.93) between the cellular activity of DT-diaphorase and hypoxic sensitivity to EO9, that is sensitivity decreases with increasing DT-diaphorase activity. Moreover, this correlation was lost when cells were exposed to drug in the presence of dicoumarol, supporting an involvement of DT-diaphorase in this relationship. These observations question the previously straightforward role for DT-diaphorase in the metabolic activation of EO9. Whereas DT-diaphorase is associated with increased toxicity in air, it appears to reduce the cytotoxicity of EO9 in hypoxic conditions. This suggests either that the one-electron reduction product of EO9 metabolism, the semiquinone, is more toxic than the two-electron reduction product, the hydroquinone, or that the hydroquinone is not cytotoxic and aerobic toxicity is due to the transient appearance of the semiquinone upon back oxidation of the hydroquinone.


*Br. J. Cancer* (1994), **70**, 1136–1143 © Macmillan Press Ltd., 1994

### DT-diaphorase protects cells from the hypoxic cytotoxicity of indoloquinone EO9

J.A. Plumb, M. Gerritsen & P. Workman

*CRC Department of Medical Oncology, University of Glasgow, Cancer Research Campaign Laboratories, Garscube Estate, Bearsden, Glasgow, G61 1BD, UK.*

**Summary** Aerobic sensitivity to indoloquinone EO9 has been shown to correlate with cellular levels of the two-electron reducing enzyme DT-diaphorase. However, little is known about the relative roles of one- and two-electron reducing enzymes in the hypoxic cytotoxicity of EO9. We have characterised a panel of 23 human tumour cell lines for both bioreductive enzyme activities and aerobic sensitivity to EO9. Eight cell lines were then selected for a comparison of aerobic and hypoxic sensitivities. Activities of DT-diaphorase showed a wide range (> 10,000-fold), while activities of the one-electron reducing cytochrome *b*5 and cytochrome P450 reductases were generally lower and showed only a 15- and 25-fold range respectively. The aerobic cytotoxicity of EO9 was clearly related to the cellular levels of DT-diaphorase ($r = 0.87$), with higher levels giving increased sensitivity, but not to the levels of one-electron reducing enzymes. In contrast, there was no relationship between sensitivity to BCNU, cisplatin or the bioreductive agent SR 4233 (tirapazamine) and activities of any of these reducing enzymes. Under hypoxic conditions sensitivity to EO9 was markedly increased in cell lines with low levels of DT-diaphorase activity, while cell lines with high levels show only a small increase in sensitivity. This is reflected by a clear correlation ($r = 0.98$) between cellular DT-diaphorase activity and the ratio of aerobic to hypoxic sensitivity to EO9. However, we have now for the first time demonstrated an inverse correlation ($r = 0.93$) between the cellular activity of DT-diaphorase and hypoxic sensitivity to EO9, that is sensitivity decreases with increasing DT-diaphorase activity. Moreover, this correlation was lost when cells were exposed to drug in the presence of dicoumarol, supporting an involvement of DT-diaphorase in this relationship. These observations question the previously straightforward role for DT-diaphorase in the metabolic activation of EO9. Whereas DT-diaphorase is associated with increased toxicity in air, it appears to reduce the cytotoxicity of EO9 in hypoxic conditions. This suggests either that the one-electron reduction product of EO9 metabolism, the semiquinone, is more toxic than the two-electron reduction product, the hydroquinone, or that the hydroquinone is not cytotoxic and aerobic toxicity is due to the transient appearance of the semiquinone upon back oxidation of the hydroquinone.

Indoloquinone EO9 is a novel bioreductive agent currently undergoing phase I/II evaluation under the auspices of the EORTC (Schellens *et al.*, 1994). It is structurally related to mitomycin C but is less myelosuppressive (Hendriks *et al.*, 1993). Mitomycin C is relatively cytotoxic under aerobic conditions and shows little increase in cytotoxicity under hypoxic conditions (Stratford & Stephens, 1989; Workman, 1992). It lacks therefore, the selectivity for hypoxic areas which are thought to be present in some solid tumours and to confer radioresistance on these cancers (Coleman, 1988; Gatenby *et al.*, 1988). While mitomycin C shows only a modest increase in activity under hypoxic conditions, sensitivity to EO9 can be increased by up to 1,000-fold (Plumb & Workman, 1994; Robertson *et al.*, 1994).

DT-diaphorase is an obligate two-electron reducing enzyme known to play a role in the detoxification of simple quinone-containing compounds (Iyanagi & Yamazaki, 1970). EO9 is a good substrate for DT-diaphorase, being reduced at about a sixth of the rate for the benchmark quinone menadione but 5,000-fold faster than mitomycin C (Walton *et al.*, 1991). Following our observation that in a pair of mouse solid colon carcinomas *in vivo* EO9 showed much greater activity against the tumour possessing high levels of DT-diaphorase and a similar relationship for two human colon carcinoma lines in culture (Walton *et al.*, 1992), a number of reports have shown a correlation between the cellular activity of DT-diaphorase and aerobic sensitivity to EO9 *in vitro* (Phillips *et al.*, 1992; Robertson *et al.*, 1992; Smitskamp-Wilms *et al.*, 1994). That sensitivity to EO9 is related to the activity of DT-diaphorase suggests that two-electron reduction represents a pathway for the activation of this bioreductive quinone which can be exploited in the clinic (Workman & Stratford, 1993), since levels of DT-diaphorase have been reported to be increased in a number of tumour types (Riley & Workman, 1992). However, the activity of DT-diaphorase is generally much greater than that of the one-electron reducing enzymes, such as cytochrome P450 reductase and cytochrome *b*5 reductase, and it is not clear precisely what level of activity of DT-diaphorase is required to induce sensitivity to EO9. Furthermore, while hypoxia is thought to be a phenomenon restricted to poorly vascularized tumours, DT-diaphorase is widely distributed throughout the body and high levels are observed in the liver, kidney and intestine (Ernster, 1967), suggesting that these tissues may be sensitive to the aerobic toxicity of EO9. Indeed, the dose-limiting toxicity of EO9 is proteinuria (Schellens *et al.*, 1994), possibly indicative of damage to the kidney following DT-diaphorase activation in this enzyme-rich organ, while the absence of myelosuppression is consistent with the very low levels of this enzyme in the bone marrow (Lewis *et al.*, 1993).

Previously, we reported that the human colon carcinoma cell line BE, which lacks DT-diaphorase activity, is relatively resistant to the aerobic toxicity of EO9 while the HT29 human colon tumour line, which has high levels of DT-diaphorase, is relatively sensitive to the drug (Walton *et al.*, 1992). However, we went on to demonstrate that under hypoxic conditions HT29 shows little increase in sensitivity to EO9 while that for BE is increased by over 1,000-fold, such that BE becomes more sensitive than HT29 (Plumb & Workman, 1994). These results suggested that in the absence of DT-diaphorase EO9 could be activated under hypoxic conditions, presumably by one-electron reduction, to a similar or even greater extent than by DT-diaphorase. Since the two cell lines are equally sensitive to a range of other cytotoxic drugs, we suggested that this could indicate that the one-electron reduction product, the semiquinone, is more cytotoxic than the two-electron reduction product, the hydroquinone. This raised a question as to the nature of the

Correspondence: J.A. Plumb.
Received 4 May 1994; and in revised form 26 July 1994.

cytotoxic intermediate of EO9 metabolism (Plumb & Workman, 1994).

In the present paper we have extended our studies on the relative roles of one- and two-electron reducing enzymes in the bioactivation of EO9 to include a panel of 23 human tumour cell lines with a range of activities of DT-diaphorase. While our results support the correlation between levels of DT-diaphorase and sensitivity to EO9 in air, we now show for the first time that DT-diaphorase appears to protect cells from the toxicity of EO9 under hypoxic conditions. We propose that the semiquinone metabolite of EO9 may be the cytotoxic entity under both aerobic and hypoxic conditions.

### Materials and methods

### *Chemicals*

*cis*-Diamminedichloroplatinum(II) (cisplatin), menadione, 3-(4.5-dimethylthiazol-2-yl)-2,5-diphenyltetrazolium bromide (MTT), cytochrome *c*, dicoumarol, NADH and NADPH were purchased from Sigma (Poole, Dorset, UK). EO9 was provided by the New Drug Development Office of the EORTC and tirapazamine (SR 4233, WIN 59075) was kindly donated by Drs M. Tracy and W.W. Lee of SRI International (Menlo Park, CA, USA). BCNU was obtained from the US National Cancer Institute.

### *Cell lines*

Details and origins of the cell lines used are shown in Table I. The breast and ovarian cell lines were grown as monolayer cultures in RPMI-1640 medium (Life Technologies, Paisley, UK) supplemented with glutamine (2 mM), fetal calf serum (10%) and, for the ovarian cell lines, insulin (0.25 units $ml^{-1}$). The small-cell lung cancer cell lines were grown as non-adherent cultures in RPMI-1640 medium with 10% fetal calf serum. The glioma and non-small-cell lung cancer cell lines were grown as adherent cultures in a mixture of Ham's F10 and Dulbecco's modified Eagle medium (50:50, Life Technologies) supplemented with glutamine (2 mM) and fetal calf serum (10%).

### *Cytotoxicity assay*

Sensitivities of the cell lines to a number of cytotoxic drugs was determined under aerobic conditions by a tetrazolium dye-based microtitration assay as described previously (Plumb *et al.*, 1989). Briefly, adherent cells were plated out at a density of $10^3$ cells per well in 96-well flat-bottomed plates (Linbro from ICN Biomedicals, High Wycombe, Bucks, UK) and allowed to attach and grow for 3 days. Non-adherent cells were plated out in a total volume of 100 µl and 100 µl of drug at twice the final concentration was added immediately. Cells were exposed to the cytotoxic drugs for 24 h and then fed with fresh medium daily for 3 days. On the fourth day, cells were fed with medium containing HEPES buffer (10 mM) and MTT (50 µl, 5 mg $ml^{-1}$) was added to each well. Plates were incubated in the dark at 37°C for 4 h, medium and MTT removed and MTT–formazan crystals dissolved in dimethylsulphoxide (200 µl per well). Glycine buffer (25 µl per well, 0.1 M, pH 10.5) was added and the absorbance measured at 570 nm in a multiwell plate reader (model 3550 EIA reader, Bio-Rad, Hemel Hempstead, Herts, UK).

A typical dose–response curve consisted of eight drug concentrations and four wells were used per drug concentration. Within an experiment triplicate determinations were made for each treatment and the three dose–response curves were obtained from separate plates. Results are expressed in terms of the drug concentration required to kill 50% of the cells ($ID_{50}$), estimated as the absorbance value equal to 50% of that of the cells in the control untreated wells.

### *Hypoxic cytotoxicity assay*

For estimation of the oxic–hypoxic cytotoxicity ratio, adherent cells were grown on glass for drug exposure and sensitivity was determined by a clonogenic assay. Cells were plated out at a density of $2 \times 10^5$ cells per 5 cm glass Petri dish and allowed to attach and grow for 2 days. The medium was removed from the dishes and replaced with 2.5 ml of fresh medium containing the drug. They were exposed to drug for 3 h in a humidified atmosphere under oxic (2% carbon dioxide in air) or hypoxic (2% carbon dioxide in nitrogen) conditions. After removal of the medium cells were detached with trypsin (0.25% trypsin + EDTA 1 mM in phosphate-buffered saline PBS) and resuspended in fresh medium. Cells from the control, untreated, dishes were

**Table I** Characteristics and sources of the 23 human tumour cell lines

| *Tumour origin* | *Cell line* | *Characteristics* | *Source* |
|---|---|---|---|
| Breast | MDAMB231 | Adenocarcinoma | ATCC |
| | ZR75 | Adenocarcinoma | ATCC |
| | MCF7 | Adenocarcinoma | Dr K. Cowan, NCI, USA |
| | MCF7/Adr | Doxorubicin resistant | |
| Glioma | GUVW | Fibroblastic | Medical Oncology, Glasgow |
| | U251 | Epithelioid | Dr B. Westermark, Uppsala |
| | SB18 | Fibroblastic | Dr J. Pilkington, Institute of Psychiatry, London |
| | GCCM | Fibroblastic | Medical Oncology, Glasgow |
| | T98G | Glioblastoma | ATCC |
| Ovary | A2780 | Adenocarcinoma | Dr R.F. Ozols, |
| | 2780CP | Cisplatin resistant | Fox Chase Cancer Centre |
| | 2780AD | Doxorubicin resistant | Pennsylvania, USA |
| | OVCAR3 | Adenocarcinoma | |
| | OVCAR4 | Adenocarcinoma | |
| | OVCAR5 | Adenocarcinoma | |
| Lung | H69 | Small-cell | ATCC |
| | H69LX10 | Doxorubicin resistant | Dr P. Twentyman MRC Cambridge |
| | H187 | Small-cell | ATCC |
| | H128 | Small-cell | ATCC |
| | CALU-3 | Adenocarcinoma | ATCC |
| | SK-MES | Squamous | ATCC |
| | LDAN | Squamous | Medical Oncology, Glasgow |
| | A549 | Type II properties | ATCC |

counted, diluted and plated out into plastic Petri dishes (6 cm, Nunclon from Life Technologies) at a density of $10^3$ cells per dish. Cells from the drug-treated dishes were diluted and plated out as for the control dishes. After incubation for 10 days at 37°C, colonies were fixed in methanol, stained with crystal violet (0.1%) and colonies of more than 50 cells counted. Drug sensitivity is expressed as the $ID_{50}$, which is the concentration required to reduce the number of colonies to 50% of that in the control, untreated, dishes.

For the non-adherent small-cell lung cancer cell lines hypoxic sensitivity was determined by MTT dye reduction (Plumb *et al.*, 1989; Stratford & Stephens, 1989). Cells were plated out in a total volume of 200 µl into glass inserts in 24-well plates at a density of $10^5$ cells $ml^{-1}$. EO9 (50 µl) was added at five times the final concentration. They were incubated for 3 h at 37°C under oxic or hypoxic conditions. The medium was then changed and cells were fed daily for the following 3 days. They were then exposed to MTT for 4 h and processed as usual except that the contents of the wells were transferred to cuvettes and absorbance at 570 nm recorded in a spectrophotometer.

*Estimation of enzyme activities*

Cells were grown in 75 $cm^2$ flasks, trypsinised if required, and washed twice with ice-cold PBS. They were resuspended in 1 ml of PBS containing aprotonin (1%), sonicated on ice and the suspension centrifuged at 4°C in an Eppendorf microfuge. DT-diaphorase activity in the supernatant was determined spectrophotometrically by following the reduction of cytochrome *c* at 550 nm using a modification of the method of Ernster (1967). A sample of the supernatant (5 µl) was added to the reaction mixture, which contained cytochrome *c* (77 µM), menadione (20 µM) or EO9 (50 µM) as the intermediate electron acceptor, NADPH (2 mM) as co-factor and bovine albumin (0.14%, w/v). Reactions were performed at 37°C in a total volume of 1 ml Tris-HCl buffer (50 mM, pH 7.4) in the presence and absence of the inhibitor dicoumarol (100 µM). DT-diaphorase activity was taken as the activity that could be inhibited by dicoumarol and is reported as nmol of cytochrome *c* (e $21.1 \times 10^3$ M $cm^{-1}$) reduced per minute per $10^6$ cells.

Cytochrome P450 reductase and cytochrome *b5* reductase activities were determined as above except that the intermediate electron acceptor was omitted from the reaction mixture and the co-factor used was NADPH for cytochrome P450 reductase and NADH for cytochrome *b5* reductase.

*Statistical analysis*

Statistically significant differences were determined by Student's *t*-test.

### Results

*Activity of DT-diaphorase, cytochrome* b5 *reductase and cytochrome P450 reductase*

The activities of DT-diaphorase, cytochrome *b5* reductase and cytochrome P450 reductase in the 23 human tumour cell lines are shown in Table II. There is a wide range (1.3–13,571 nmol $min^{-1}$ $10^6$ cells, > 10,000-fold) of activities of DT-diaphorase, with the most marked difference observed between the small-cell and non-small-cell lung cancer cell lines. However, within each tumour type there is a range of activities. In contrast, activities of cytochrome *b5* reductase and cytochrome P450 reductase show only a 15 (11.5–170.5 nmol $min^{-1}$ $10^{-6}$ cells) and 25 (5.2–127.5 nmol $min^{-1}$ $10^{-6}$ cells) fold range respectively. these two enzyme activities are lower than those of DT-diaphorase except in the case of seven of the cell lines in which the activity of DT-diaphorase is less than 35 nmol $min^{-1}$ $10^{-6}$ cells. Although the two drug-resistant variants of A2780 show significantly increased activities of DT-diaphorase (2780AD, 1.9-fold, $P<0.01$; 2780CP, 3.6-fold, $P<0.01$), this is not the case for MCF7/Adr and H69LX10 compared with their parent lines.

*Aerobic drug sensitivities of the cell lines*

Sensitivities of the cell lines to EO9, SR 4233, BCNU and cisplatin are also shown in Table II. As shown clearly in Figure 1 there is a correlation ($r = 0.87$) between sensitivity to EO9 and the cellular activity of two-electron reducing

**Table II** Activities (nmol $min^{-1}$ $10^{-6}$ cells) of DT-diaphorase, cytochrome *b5* reductase and cytochrome P450 reductase in the human tumour cell lines. Also shown are the sensitivites of the cell lines to EO9, SR 4233, BCNU and cisplatin. Sensitivity was determined by an MTT-based cytotoxicity assay. Cells were exposed to drug for 24 h and sensitivity is expressed as the $ID_{50}$ concentration. Results are the mean ± s.e.m. of triplicate estimations

| | | *Enzyme activity (nmol min⁻¹ 10⁻⁶ cells)* | | | *ID₅₀* | | | |
|---|---|---|---|---|---|---|---|---|
| *Tumour* | *Cell line* | *DT-diaphorase* | *b5 reductase* | *P450 reductase* | *EO9 (nM)* | *SR4233 (µM)* | BCNU (*µM*) | *Cisplatin (µM)* |
| Breast | MDAMB231 | 6 ± 4 | 105.0 ± 11.6 | 101.5 ± 4.6 | 320.0 ± 11.5 | 290.0 ± 2.7 | 55.3 ± 2.3 | 5.0 ± 0.4 |
| | ZR75 | 1096 ± 144 | 40.0 ± 1.3 | 31.0 ± 2.0 | 32.0 ± 3.1 | 1400 ± 77 | 188.3 ± 21.3 | 8.7 ± 0.4 |
| | MCF7 | 4476 ± 89 | 37.0 ± 2.6 | 29.0 ± 1.7 | 4.0 ± 0.3 | 65.0 ± 4.8 | 88.0 ± 10.4 | 1.2 ± 0.1 |
| | MCF7/Adr | 35 ± 2 | 50.5 ± 3.0 | 35.5 ± 3.9 | 55.0 ± 2.9 | 940.0 ± 97.0 | 251.7 ± 1.7 | 6.1 ± 0.2 |
| Glioma | GUVW | 166 ± 31 | 119.5 ± 16.0 | 70.0 ± 2.6 | 150.0 ± 11.5 | 205.0 ± 25.5 | 38.0 ± 3.5 | 0.8 ± 0.1 |
| | U251 | 298 ± 80 | 102.0 ± 4.1 | 74.5 ± 5.4 | 2.0 ± 0.2 | 42.5 ± 9.2 | 18.2 ± 1.1 | 0.08 ± 0.003 |
| | SB18 | 685 ± 81 | 80.5 ± 4.9 | 54.0 ± 2.2 | 25.0 ± 1.3 | 593.0 ± 60.0 | 61.0 ± 11.6 | 0.7 ± 0.2 |
| | GCCM | 961 ± 323 | 170.5 ± 4.5 | 127.5 ± 8.8 | 22.0 ± 1.2 | 708.5 ± 124.5 | 59.3 ± 2.0 | 1.6 ± 0.3 |
| | T98G | 9334 ± 547 | 106.5 ± 6.2 | 73.5 ± 3.4 | 10.0 ± 1.0 | 160.0 ± 6.0 | 221.7 ± 19.2 | 3.9 ± 0.2 |
| Ovary | OVCAR4 | 225 ± 108 | 73.0 ± 2.8 | 39.0 ± 3.9 | 180.0 ± 21.9 | 266.5 ± 24.5 | 102.7 ± 4.1 | 2.3 ± 0.3 |
| | A2780 | 554 ± 38 | 36.5 ± 7.0 | 15.5 ± 0.7 | 2.0 ± 0.1 | 4.5 ± 0.6 | 9.7 ± 1.0 | 0.2 ± 0.01 |
| | 2780AD | 1023 ± 40 | 18.0 ± 0.7 | 12.5 ± 0.8 | 10.0 ± 4.4 | 74.5 ± 10.7 | 33.0 ± 1.2 | 1.1 ± 0.1 |
| | OVCAR3 | 1768 ± 202 | 128.0 ± 8.4 | 111.5 ± 9.6 | 16.0 ± 2.7 | 68.5 ± 12.9 | 225.0 ± 40.1 | 0.8 ± 0.04 |
| | 2780CP | 2014 ± 225 | 41.0 ± 2.5 | 25.0 ± 2.3 | 14.0 ± 0.4 | 44.5 ± 3.5 | 49.7 ± 2.9 | 3.5 ± 0.2 |
| | OVCAR5 | 4109 ± 195 | 87.0 ± 5.8 | 69.5 ± 4.8 | 13.0 ± 1.2 | 77.5 ± 8.2 | 113.3 ± 4.4 | 1.5 ± 0.3 |
| Lung | H69LX10 | 1.3 ± 0.5 | 53.0 ± 2.1 | 35.0 ± 1.5 | 2450 ± 148 | 46.5 ± 7.0 | 4.6 ± 0.6 | 1.5 ± 0.2 |
| | H69 | 2.0 ± 1.0 | 61.5 ± 4.8 | 74.0 ± 3.4 | 2467 ± 87 | 13.5 ± 3.6 | 5.5 ± 1.2 | 2.2 ± 0.6 |
| | H187 | 6.6 ± 0.9 | 12.2 ± 0.4 | 5.5 ± 0.1 | 425.0 ± 128.0 | 107.0 ± 10.2 | 7.0 ± 0.4 | 0.8 ± 0.1 |
| | H128 | 9.9 ± 1.4 | 11.5 ± 0.5 | 5.2 ± 0.1 | 646.0 ± 104.0 | 167.0 ± 68.0 | 174.0 ± 54.9 | 8.0 ± 2.4 |
| | CALU-3 | 30 ± 3 | 109.5 ± 9.5 | 64.0 ± 7.6 | 97.0 ± 8.7 | 59.0 ± 3.9 | 158.3 ± 3.3 | 4.3 ± 0.2 |
| | SK-MES | 1849 ± 103 | 158.0 ± 18.7 | 67.0 ± 9.7 | 8.0 ± 0.3 | 110.0 ± 8.1 | 2.6 ± 0.4 | 2.9 ± 0.3 |
| | LDAN | 2066 ± 140 | 105.5 ± 14.4 | 74.5 ± 2.0 | 12.0 ± 0.4 | 58.5 ± 4.6 | 151.7 ± 10.9 | 3.5 ± 0.1 |
| | A549 | 13571 ± 1365 | 115.5 ± 5.5 | 79.5 ± 9.0 | 4.0 ± 0.3 | 98.0 ± 12.3 | 113.4 ± 8.3 | 2.7 ± 0.2 |

DT-diaphorase. Increased sensitivity to EO9 is associated with elevated levels of DT-diaphorase activity. No such correlation is apparent for SR 4233 ($r = 0.07$), BCNU ($r = 0.39$) and cisplatin ($r = 0.10$). Furthermore, there is no relationship between the activities of the one-electron reducing enzymes, cytochrome *b*5 reductase ($r = 0.26$) and cytochrome P450 reductase ($r = 0.19$), and sensitivity to EO9 or to any of the other three drugs (Table II). Of note, two of the cell lines, U251 and A2780, are markedly more sensitive to EO9 than is predicted from their levels of DT-diaphorase activity (Figure 1, Table II). However, these two cell lines are also the most sensitive to cisplatin and both are relatively sensitive to BCNU and SR 4233, suggesting an overall sensitivity to DNA-damaging agents. The drug-resistant variants of A2780 are cross-resistant (by 5 to 7-fold) to EO9, as is the doxorubicin-resistant breast cell line MCF7/Adr (14-fold), but this is not the case for the doxorubicin-resistant small-cell lung cancer cell line H69LX10. For 2780AD and 2780CP this cross-resistance correlates with increased activities of DT-diaphorase.

### *Sensitivity to EO9 under hypoxic conditions*

For each tumour type sensitivity to EO9 under hypoxic conditions was determined for the cell lines with the highest and lowest levels of DT-diaphorase activity (Table III). DT-diaphorase activity was determined with either menadione or EO9 as the intermediate electron acceptor. Activities measured with EO9 as the substrate were much lower than those for menadione, but the relative activities for the eight cell lines were consistent for the two substrates ($r = 0.97$, Figure 2).

For each pair of cell lines within a given tumour type, the increase in sensitivity to EO9 under hypoxic conditions was greatest for the cell line with lowest level of DT-diaphorase activity (Table IV). This is, for example, most obvious in the two breast lines, in which the enzyme-rich MCF7 cell line gave an increase in sensitivity of only 2-fold, whereas the low DT-diaphorase line MDAMB231 exhibited an increase of 757-fold. Note that for the cells showing the greatest enzyme activity, the A549 lung cancer line, there was no change at all in sensitivity to EO9 under hypoxic compared with aerobic conditions. Furthermore, there was a clear correlation ($r = 0.98$) between DT-diaphorase activity and the aerobic–hypoxic cytotoxicity ratio (Table IV, Figure 3). Again as seen for the larger series, under aerobic conditions, the cell lines with the highest levels of DT-diaphorase activity were most sensitive to EO9 ($r = 0.95$, Figure 4a). Importantly, however, under hypoxic conditions these cell lines were the least sensitive to EO9 and the reverse correlation was seen ($r = 0.93$, Figure 4b).

### *Effect of dicoumarol on the aerobic and hypoxic toxicity of EO9*

Sensitivity to EO9 was also determined in the presence of dicoumarol (200 μM), an inhibitor of DT-diaphorase (Table III). This concentration had no effect on cell survival. Dicoumarol decreased the aerobic sensitivity of all eight cell lines by 2.5- to 5.1-fold (Table IV) but the correlation between sensitivity to EO9 and activity of DT-diaphorase remained (Figure 4a). In contrast, the hypoxic sensitivity of seven of the cell lines was *increased* in the presence of dicoumarol, and the increase was greatest for the cell lines

**Figure 1** Activity of DT-diaphorase, determined with menadione as the intermediate electron acceptor, and sensitivity to EO9 ($r = 0.87$) for the 23 human tumour cell lines. Results are those presented in Table II.

**Figure 2** Relationship ($r = 0.97$) between the activity of DT-diaphorase determined with menadione as the intermediate electron acceptor and that determined with EO9. Results are shown for the four pairs of the cell lines and are those presented in Table III.

**Table III** Activities of DT-diaphorase and sensitivity to EO9 determined in the presence (aerobic) and absence (hypoxic) of air for four pairs of cell lines selected as those with the highest and lowest levels of DT-diaphorase activity from each of the four tumour types. DT-diaphorase activity (nmol min$^{-1}$ 10$^{-6}$ cells) was determined with either menadione or EO9 as the intermediate electron acceptor. Sensitivity to EO9 (ID$_{50}$) was determined by a clonogenic assay and cells were exposed to drug for 3 h. Also shown is the sensitivity to EO9 determined in the presence of dicoumarol (200 μM). Results are the mean ± s.e.m. of triplicate estimations

| | | *DT-diaphorase (nmol min$^{-1}$ 10$^{-6}$ cells)* | | *ID$_{50}$ (nM) EO9* | | *ID$_{50}$ (nM) EO9 + dicoumarol* | |
|---|---|---|---|---|---|---|---|
| *Tumour* | *Cell line* | *Menadione* | *EO9* | *Aerobic* | *Hypoxic* | *Aerobic* | *Hypoxic* |
| Breast | MDAMB231 | 6 ± 4 | 1.5 ± 0.3 | 18167 ± 1202 | 24 ± 7 | 60333 ± 3180 | 9 ± 3 |
| | MCF7 | 4476 ± 89 | 57.0 ± 2.0 | 166 ± 11 | 81 ± 16 | 532 ± 141 | 6 ± 2 |
| Glioma | GUVW | 166 ± 31 | 15.0 ± 2.6 | 2050 ± 226 | 38 ± 10 | 9400 ± 1137 | 22 ± 7 |
| | T98G | 9334 ± 547 | 113.5 ± 6.6 | 290 ± 20 | 117 ± 26 | 1467 ± 83 | 33 ± 11 |
| Ovary | OVCAR4 | 225 ± 108 | 13.0 ± 0.6 | 1983 ± 169 | 49 ± 2 | 7967 ± 328 | 21 ± 6 |
| | OVCAR5 | 4109 ± 195 | 50.5 ± 3.6 | 337 ± 3 | 58 ± 11 | 1673 ± 23 | 8 ± 2 |
| Lung | CALU-3 | 30 ± 3 | 10.0 ± 0.3 | 2085 ± 43 | 32 ± 12 | 5233 ± 657 | 30 ± 8 |
| | A549 | 13571 ± 1365 | 193.5 ± 15.2 | 238 ± 30 | 233 ± 48 | 590 ± 25 | 37 ± 9 |

**Table IV** Hypoxic cytotoxicity ratio for EO9 (aerobic $ID_{50} \div$ hypoxic $ID_{50}$) in the eight cell lines shown in Table III. Also shown is the *decrease* in aerobic and *increase* in hypoxic sensitivity to EO9 in the presence of dicoumarol

| | | $ID_{50}$ EO9 | | |
|---|---|---|---|---|
| *Tumour* | *Cell line* | *Aerobic / hypoxic* | *Aerobic + dicoumarol / aerobic* | *Hypoxic / hypoxic + dicoumarol* |
| Breast | MDAMB231 | 757.0 | 3.3 | 2.7 |
| | MCF7 | 2.1 | 3.2 | 13.5 |
| Glioma | GUVW | 54.0 | 4.6 | 1.7 |
| | T98G | 2.5 | 5.1 | 3.6 |
| Ovary | OVCAR4 | 40.5 | 4.0 | 2.3 |
| | OVCAR5 | 5.8 | 5.0 | 7.3 |
| Lung | CALU-3 | 65.2 | 2.5 | 1.1 |
| | A549 | 1.0 | 2.5 | 6.3 |

**Figure 3** Correlation between the activity of DT-diaphorase and the hypoxic cytotoxicity ratio for EO9 ($r = 0.98$). DT-diaphorase activity was determined with EO9 as intermediate electron acceptor and the hypoxic sensitisation ratio is the sensitivity of the cell line to EO9 in air divided by the hypoxic sensitivity to EO9. Results are those shown in Table III.

**Figure 4** DT-diaphorase activity and sensitivity to EO9 in the presence (O) and absence (●) of dicoumarol (200 μM) determined under both aerobic (**a**)'and hypoxic (**b**) conditions. Results are shown for the four pairs of cell lines and are those presented in Table III.

with the highest levels of DT-diaphorase activity (Table IV). In the presence of dicoumarol there was no relationship between DT-diaphorase activity and the hypoxic sensitivity to EO9 (Figure 4b).

### Discussion

We have confirmed, in a large panel of breast, glioma, ovarian and lung tumour cell lines, a clear correlation between cellular levels of DT-diaphorase and sensitivity in air to EO9 (Table II, Figure 1). For cell lines that have low activities of DT-diaphorase, sensitivity to EO9 is markedly increased by exposure to drug in the absence of air. In contrast, cell lines with high levels of DT-diaphorase show little increase in sensitivity to EO9 under hypoxic conditions. As a consequence, the degree of sensitisation is inversely proportional to the cellular activity of DT-diaphorase (Figure 3). More importantly, we have shown for the first time that under hypoxic conditions the correlation is reversed such that DT-diaphorase appears to protect cells from the cellular toxicity of EO9 (Figure 4b).

It is apparent from this panel of tumour cell lines that activities of DT-diaphorase vary widely (Table II), but it is not known if this is a true reflection of the activities present in the tumours from which the lines were derived. Activities are uniformly low in all the small-cell lung cancer cell lines, but cell lines with low activities were present in each of the tumour types. The difference between small-cell and non-small-cell lung cancer cell lines has been reported previously, and this did seem to reflect activities observed in lung tumour samples (Malkinson *et al.*, 1992). There was a much smaller range of activities of the two one-electron reducing enzymes (Table II). The cell lines showed a wide range of sensitivities to SR 4233, BCNU and cisplatin, but these sensitivities did not relate to the activities of any of the three enzyme studied. This is not surprising for BCNU and cisplatin since there is no evidence that for cytotoxicity these agents require metabolic activation. The benzotriazine di-N-oxide SR 4233 (tirapazamine) is a poor substrate for DT-diaphorase, and two-electron reduction has been proposed to inactivate this bioreductive agent (Walton & Workman, 1990; Brown, 1991). The lack of a correlation between DT-diaphorase activity and sensitivity to SR 4233 suggests that DT-diaphorase does not play a major role in the cellular detoxification of this drug, and this supports our previous observation (Plumb & Workman, 1994). In contrast, the present results suggest that DT-diaphorase plays a key role in the aerobic cytotoxicity of EO9, and this supports previous observations (Phillips *et al.*, 1992; Robertson *et al.*, 1992; Walton *et al.*, 1992; Plumb & Workman, 1994; Smitskamp-Wilms *et al.*, 1994).

**Figure 5** Possible routes of bioactivation of EO9 to DNA reactive species by one- and two-electron reducing enzymes.

It is thought that one-electron reduction of EO9 results in the production of a semiquinone free radical (Figure 5). In air it would be expected that this radical is rapidly back-oxidised with the production of oxygen radicals which, although toxic, can be removed by cellular detoxifying enzymes. However, rapid redox cycling itself can cause cell death owing to depletion of reduced co-factors (Workman, 1992). Two-electron reduction of quinones by DT-diaphorase is thought to produce a potentially more stable hydroquinone (Pan *et al.*, 1984), and for EO9 it has been proposed that this can alklyate DNA, giving rise to DNA strand breaks and cross-links which have been reported (Walton *et al.*, 1991; Bailey *et al.*, 1994*a*). From a chemical perspective, either one- or two-electron reduction would be predicted to activate electrophilic sites in the EO9 molecule. It would be expected that cell lines will differ in their ability to withstand DNA damage, and this is reflected in the range of sensitivities to cisplatin and to the alkylating agent BCNU (Table II). In view of this additional source of variability, it is perhaps surprising that such a clear correlation between DT-diaphorase activity and EO9 sensitivity is observed (Figure 1) unless toxicity is related not to specific DNA adduct formation but to some other type of DNA damage such as strand breaks. Two cell lines (A2780 and U251) are more sensitive to EO9 than would be predicted from DT-diaphorase activities alone. However, these cell lines are also very sensitive to the other three drugs used, which suggests that they may be inherently sensitive to DNA-damaging agents (Table II).

A number of agents, including cytotoxic drugs, are known to induce expression of DT-diaphorase (Prochaska & Talalay, 1988), and it might be expected that cell lines made drug resistant *in vitro* would show increased activity. This is true for the doxorubicin (2780AD)- and cisplatin (2780CP)-resistant ovarian cell lines, and the results are consistent with the observed decrease in aerobic sensitivity to EO9 (Table II). However, the elevation appears to be specific to this model since the doxorubicin-resistant small-cell lung cancer (H69LX10) cell line does not show increased activity of DT-diaphorase and the breast cell line MCF7/Adr has a marked decrease in activity which is associated with a reduction in sensitivity to EO9 (Table II).

In order to compare the relative roles of DT-diaphorase and hypoxia in the metabolic activation of EO9, we selected four representative pairs of cell lines from the panel. The cell lines with the highest and lowest activities of DT-diaphorase from each tumour type were used. DT-diaphorase activity is commonly determined with the natural quinone menadione as the substrate. However, we had two concerns with respect to the sole reliance on using menadione as the substrate in these studies. Firstly, while menadione is a good substrate for DT-diaphorase from both rat and human, EO9 is a better substrate for the rat enzyme (Walton *et al.*, 1991). Secondly, these cell lines are all derived from tumours and genetic mutations are known to alter the catalytic behaviour of the enzyme (Chen *et al.*, 1992; Ma *et al.*, 1992). Thus, for these eight cell lines DT-diaphorase activity was determined with EO9 as well as menadione as the substrate. In all cases activity was higher for menadione than EO9 (Table III). However, although the relative activities when comparing cell lines showed some variability depending on the substrate used, the overall rank order was not changed (Figure 2).

In contrast to the one-electron reduction reaction, the two-electron reduction reaction catalysed by DT-diaphorase is generally regarded as oxygen independent. Hence, EO9 can be metabolised by both one- and two-electron reduction reactions, but the balance between these pathways will be determined by both the relative activities of one- and two-electron reducing enzymes and by the presence or absence of oxygen (Figure 5). Our results suggest that both pathways are important in the activation of EO9 since the degree of enhancement of cytotoxicity under hypoxic conditions is related to the cellular activity of DT-diaphorase (Figure 3). Thus, in cell lines with high levels of DT-diaphorase activity EO9 is activated to a cytotoxic species in air (Table III). In contrast, in cell lines with low levels of DT-diaphorase little activation is observed in air but EO9 can be activated by one-electron reduction under hypoxic conditions. This supports our previous observations with the pair of colon carcinoma cell lines in which a substantial increase in sensitivity to EO9 under hypoxic conditions was observed only in the BE cell line, which lacked DT-diaphorase activity (Plumb & Workman, 1994). Although EO9 is not as good as menadione as a substrate for DT-diaphorase (Table III), this enzyme appears to be a potent activator of EO9 in cells since the large hypoxic sensitisation ratios are only observed in cell lines with negligible levels of activity such as BE (Plumb & Workman, 1994) or MDAMB231 (Table III). This may be an important observation in terms of potential toxicity to normal tissues. Although DT-diaphorase activity may be elevated in tumours it is possible that levels in some normal tissues are already sufficient for significant aerobic activation of EO9.

Our results show that, in contrast to the aerobic situation, under hypoxic conditions sensitivity to EO9 is inversely correlated with DT-diaphorase activity such that the cell lines with the highest levels of DT-diaphorase are the least sensitive (Figure 4b). The implication is that DT-diaphorase protects from the hypoxic toxicity of EO9, and this is supported by the observation that this protection is lost in the presence of dicoumarol, an inhibitor of DT-diaphorase. There are problems associated with the use of dicoumarol as a specific inhibitor of DT-diaphorase since it is known to have other effects including inhibition of cytochrome *b*5 reductase (Komiyama *et al*., 1982; Workman *et al*., 1989). Enhanced hypoxic toxicity of mitomycin C in the presence of dicoumarol has been observed (Keyes *et al*., 1985), and it was suggested that this was due to increased activity of xanthine dehydrogenase, which catalyses a two-electron reduction reaction (Gustafson & Pritsos, 1992; Bizanek *et al*., 1993). The clear correlation between sensitivity to EO9 and activity of DT-diaphorase in air indicates that xanthine dehydrogenase makes a negligible contribution to the activation of EO9 unless it shows the same activity profile as DT-diaphorase. Dicoumarol decreased the aerobic toxicity of EO9 (Figure 4a), which suggests that in air at least the major effect is inhibition of DT-diaphorase. Furthermore, dicoumarol increased the hypoxic toxicity of EO9 in DT-diaphorase-rich HT29 cells but not BE cells, which express a mutant enzyme that lacks detectable activity (Plumb & Workman, 1994), and in both cell lines activity of xanthine oxidase is undetectable (Siegel *et al*., 1990). Xanthine dehydrogenase is probably the major form of the enzyme in cells but it is rapidly converted to one-electron reducing xanthine oxidase on isolation (Gustafson & Pritsos, 1992). We do not think that increased activity of xanthine dehydrogenase accounts for the increased hypoxic toxicity of EO9 but suggest that dicoumarol overcomes the previously unreported protective effects of DT-diaphorase.

Although our results indicate a role for DT-diaphorase in the aerobic toxicity of EO9, it is not necessarily the hydroquinone that is responsible for the observed aerobic toxicity. EO9 shows significant toxicity in air even in cell line BE, which lacks DT-diaphorase activity. This suggests either that the parent drug is itself cytotoxic or that generation of drug or oxygen radicals through one-electron reduction makes a significant contribution to cell kill. We have shown that even a small activity of DT-diaphorase significantly increases the aerobic cytotoxicity of EO9 (H187 and H128 cf H69, Table II). It is unlikely that low activities will rapidly deplete the pool of parent drug in cells, and this observation would argue in favour of toxicity being due to metabolism of the drug. The hydroquinone product of DT-diaphorase activity can, in theory, be back-oxidised, via the semiquinone, to EO9 in air but not under hypoxic conditions (Figure 5). Since DT-diaphorase is protective under hypoxic conditions, this suggests that the hydroquinone is less toxic than the semiquinone, and this has also been proposed for mitomycin C (Dulhanty & Whitmore, 1991). From a chemical perspective it might be predicted that formation of the semiquinone free radical would activate the aziridine group together with the two alkylating vinylogous side chains more effectively than would be the case for the hydroquinone metabolite. However, our results would also support the proposal that the hydroquinone is not cytotoxic and that aerobic toxicity is the result of oxygen radical formation and transient appearance of the semiquinone. This is supported by the identification by electron spin resonance of a drug-based free radical, presumably the semiquinone, when EO9 is metabolised under aerobic conditions by either cytochrome P450 reductase or DT-diaphorase (Bailey *et al*., 1993). In addition, metabolism of EO9 by either DT-diaphorase or cytochrome P450 reductase has been shown to produce DNA strand breaks (Walton *et al*., 1991; Bailey *et al*., 1994*b*). We propose that, since in this panel of cell lines activities of the one-electron reducing enzymes are generally low compared with that of DT-diaphorase substantial formation and persistence of the semiquinone will only occur in those with sufficient DT-diaphorase activity. Although the semiquinone is unstable in air the 1,000-fold hypoxic sensitisation ratio reported for BE (Plumb & Workman, 1994) and the 757-fold ratio for the breast cell line MDAMB231 reported here indicates that the semiquinone must be a highly potent cytotoxin.

Given the very promising preclinical and early clinical results seen with EO9 it is clearly important to understand the mechanisms involved in the activation of the drug. Agents such as SR 4233 that are activated only under hypoxic conditions have limited use as single agents since they target a small subpopulation of the tumour. Because EO9 is activated by both one- and two-electron reduction, it can potentially target both aerobic and hypoxic areas of the tumour. However, if DT-diaphorase protects hypoxic cells from the toxicity of EO9 the drug cannot be used to target both oxic and hypoxic areas of the same tumour.

We thank the Cancer Research Campaign for financial support. Paul Workman acknowledges the award of a CRC Life Fellowship.

### References

BAILEY, S.M., LEWIS, A.D., PATTERSON, L.H., FISHER, G.R. & WORKMAN, P. (1993). Free radical generation following reduction of EO9: involvement in cytotoxicity. *Br. J. Cancer*, **67** (Suppl. 20), 8.

BAILEY, S.M., WYATT, M.D., LEWIS, A.D., HARTLEY, J.A. & WORKMAN, P. (1994*a*). Involvement of DT-diaphorase in the DNA cross-linking and sequence selectivity of the novel indoloquinone antitumour agent EO9. *Proc. Am. Ass. Cancer Res*., **35**, 384.

BAILEY, S.M., LEWIS, A.D. & WORKMAN, P. (1994*b*). Involvement of NADPH: cytochrome P450 reductase in activation of EO9 to a DNA damaging species. *Br. J. Cancer*, **69** (Suppl. 21), 57.

BIZANEK, R., CHOWDARY, D., ARAI, H., KASAI, M., HUGHES, C.S., SARTORELLI, A.C., ROCKWELL, S. & TOMAZ, M. (1993). Adducts of mitomycin C in EMT6 mouse mammary tumor cells: effects of hypoxia and dicoumarol on adduct patterns. *Cancer Res*., **53**, 5127-5134.

BROWN, J.M. (1991). Redox activation of benzotriazine di-N-oxides: mechanisms and potential as anticancer drugs. In *Selective Activation of Drugs by Redox Processes*, Adams, G.E., Breccia, A., Fielden, E.M. & Wardman, P. (eds) pp. 137-148. Plenum: New York.

CHEN, H.-H., MA, J.-X., FORREST, G.L., DENG, P.S.K., MARTINO, P.A., LEE, T.D. & CHEN, S. (1992). Expression of rat liver NAD(P)H: quinone-acceptor oxidoreductase in *Escherichia coli* and mutagenesis *in vitro* at Arg-177. *Biochem. J*., **284**, 855-860.

COLEMAN, C.N. (1988). Hypoxia in tumours: a paradigm for the approach to biochemical and physiological heterogeneity. *J. Natl Cancer Inst*., **80**, 310-317.

DULHANTY, A.M. & WHITMORE, G.F. (1991). Chinese hamster ovary cell lines resistant to mitomycin C under aerobic but not hypoxic conditions are deficient in DT-diaphorase. *Cancer Res*., **51**, 1860-1865.

ERNSTER, L. (1967). DT-diaphorase. *Methods Enzymol*., **10**, 309-317.

GATENBY, R.A., KESSLER, H.B., ROSENBLUM, J.S., COIA, L.R., MOLDOFSKY, P.J., HARTZ, W.H. & BRODER, G.J. (1988). Oxygen distribution in squamous cell carcinoma metastases and its relationship to the outcome of radiation therapy. *Int. J. Radiat. Oncol. Biol. Phys*., **14**, 831-838.

GUSTAFSON, D.L. & PRITSOS, C.A. (1992). Bioactivation of mitomycin C by xanthine dehydrogenase from EMT6 mouse mammary carcinoma tumours. *J. Natl Cancer Inst*., **84**, 1180-1185.

HENDRIKS, H.R., PIZAO, P.E., BERGER, D.P., KOOISTRA, K., BIBBEY, M.C., BOVEN, E., DREEF-VAN DER MEULEN, H.C., HENRAR, R.E.C., FIEBIG, H.H., DOUBLE, J.A., HORNSTRA, H.W., PINEDO, H.M. & WORKMAN, P. (1993). EO9, a novel bioreductive alkylating indoloquinone with preferential solid tumour activity and lack of bone marrow toxicity in preclinical models. *Eur. J. Cancer*, **29A**, 897-906.

IYANAGI, T. & YAMAZAKI, I. (1970). One-electron transfer reactions in biochemical systems. V. Difference in the mechanism of quinone reduction by the NADH dehydrogenase and the NAD(P)H ·dehydrogenase (DT-diaphorase). *Biochim. Biophys. Acta*, **216**, 282–294.

KEYES, S.R., ROCKWELL, S. & SARTORELLI, A.C. (1985). Enhancement of mitomycin C cytotoxicity to hypoxic tumour cells by dicoumarol *in vivo* and *in vitro*. *Cancer Res.*, **45**, 213–216.

KOMIYAMA, T., KIKUCHI, T. & SUGIURA, Y. (1982). Generation of hydroxyl radical by anticancer quinone drugs carbazilquinone, mitomycin C, aclacinomycin A and adriamycin in the presence of NADPH cytochrome P450 reductase. *Biochem. Pharmacol.*, **31**, 3651–3656.

LEWIS, A.D., HOLYOAKE, T.L., DUNLOP, D.J., PRAGNELL, I.D. & WORKMAN, P. (1993). Lack of myelosuppression with EO9 in human and mouse bone marrow: correlation with low DT-diaphorase. *Proc. Am. Assoc. Cancer Res.*, **34**, 345.

MA, Q., CUI, K., WANG, R.W., LU, A.Y.H. & YANG, C.S. (1992). Site-directed mutagenesis of rat liver NAD(P)H: quinone oxidoreductase: roles of lysine 76 and cysteine 179. *Arch. Biochem. Biophys.*, **294**, 434–439.

MALKINSON, A.M., SIEGEL, D., FORREST, G.L., GAZDAR, A.F., OIE, H.K., CHAN, D.C., BUNN, P.A., MABRY, M., DYKES, D.J., HARRISON, S.D. & ROSS, D. (1992). Elevated DT-diaphorase activity and messenger RNA content in human non-small cell lung carcinoma: relationship to the response of lung tumour xenografts to mitomycin C. *Cancer Res.*, **52**, 4752–4757.

PAN, S., ANDREWS, P.A., GLOVER, C.J. & BACHUR, N.R. (1984). Reductive activation of motomycin C and mitomycin C metabolites catalysed by NADPH-cytochrome P-450 reductase and xanthine oxidase. *J. Biol. Chem.*, **259**, 959–966.

PHILLIPS, R.M., HULBERT, P.B., BIBBEY, M.C., SLEIGH, N.R. & DOUBLE, J.A. (1992). In vitro activity of the novel indoloquinone EO-9 and the influence of pH on cytotoxicity. *Br. J. Cancer*, **65**, 359–364.

PLUMB, J.A. & WORKMAN, P. (1994). Unusually marked hypoxic sensitisation to indoloquinone EO9 and mitomycin C in a human colon-tumour cell line that lacks DT-diaphorase activity. *Int. J. Cancer*, **56**, 134–139.

PLUMB, J.A., MILROY, R. & KAYE, S.B. (1989). Effects of the pH dependence of 3-(4,5-dimethylthiazol-2-yl)-2,5-diphenyltetrazolium bromide-formazan absorption on chemosensitivity determined by a novel tetrazolium-based assay. *Cancer Res.*, **49**, 4435–4440.

PROCHASKA, H.J. & TALALAY, P. (1988). Regulatory mechanisms of monofunctional and bifunctional anticarcinogenic enzyme inducers in murine liver. *Cancer Res.*, **48**, 4776–4782.

RILEY, R.J. & WORKMAN, P. (1992). DT-diaphorase and cancer chemotherapy. *Biochem. Pharmacol.*, **43**, 1657–1669.

ROBERTSON, N., STRATFORD, I.J., HOULBROOK, S., CARMICHAEL, J. & ADAMS, G. (1992). The sensitivity of human tumour cells to quinone bioreductive drugs: what role for DT-diaphorase? *Biochem. Pharmacol.*, **44**, 409–412.

ROBERTSON, N., HAIGH, A., ADAMS, G.E. & STRATFORD, I.J. (1994). Factors affecting sensitivity to EO9 in rodent and human tumour cells in vitro: DT-diaphorase activity and hypoxia. *Eur. J. Cancer Clin. Oncol.* (in press).

SCHELLENS, J.H.M., STOTER, G. & VERWEIJ, J. (1994). Limited sampling model for EO9, a novel indoloquinone cytotoxic drug. *Ann. Oncol.* (in press).

SIEGEL, D., GIBSON, N.W., PREUSCH, P.C. & ROSS, D. (1990). Metabolism of diaziquone by NAD(P)H: (quinone acceptor) oxidoreductase (DT-diaphorase): role of diaziquone-induced DNA damage and cytotoxicity in human colon carcinoma cells. *Cancer Res.*, **50**, 7293–7300.

SMITSKAMP-WILMS, E., PETERS, G.J., PINEDO, H.M., VAN ARK-OTTE, J. & GIACCONE, G. (1994). Chemosensitivity to the indoloquionone EO9 is correlated with DT-diaphorase activity and its gene expression. *Biochem. Pharmacol.*, **47**, 1325–1332.

STRATFORD, I.J. & STEPHENS, M.A. (1989). The differential hypoxic cytotoxicity of bioreductive agents determined in vitro by the MTT assay. *Int. J. Radiat. Oncol. Biol. Phys.*, **16**, 973–976.

WALTON, M.I. & WORKMAN, P. (1990). Enzymology of the reductive bioactivation of SR 4233. A novel benzotriazine di-N-oxide hypoxic cell cytotoxin. *Biochem. Pharmacol.*, **39**, 1735–1742.

WALTON, M.I., SMITH, P.J. & WORKMAN, P. (1991). The role of NAD(P)H: quinone reductase (EC 1.6.99.2, DT-diaphorase) in the reductive bioactivation of the novel indoloquinone antitumour agent EO9. *Cancer Commun.*, **3**, 199–206.

WALTON, M.I., BIBBEY, M.C., DOUBLE, J.A., PLUMB, J.A. & WORKMAN, P. (1992). DT-diaphorase activity correlates with sensitivity to the indoloquinone EO9 in mouse and human colon carcinomas. *Eur. J. Cancer Clin. Oncol.*, **28A**, 1597–1600.

WORKMAN, P. (1992). Keynote address: bioreductive mechanisms. *Int. J. Radiat. Oncol. Biol. Phys.*, **22**, 631–637.

WORKMAN, P. & STRATFORD, I.J. (1993). The experimental development of bioreductive drugs and their role in cancer therapy. *Cancer Metastasis Rev.*, **12**, 73–82.

WORKMAN, P., WALTON, M.I., POWIS, G. & SCHLAGER, J.J. (1989). DT-diaphorase: questionable role in mitomycin C resistance, but a taret for novel bioreductive drugs. *Br. J. Cancer*, **60**, 800.